# Isolated Retropancreatic Tuberculous Lymphadenitis Mimicking Carcinoma: A Diagnostic Challenge

**DOI:** 10.1155/2016/7295496

**Published:** 2016-05-30

**Authors:** H. Kuriry, R. Alenezi, A. Alghamdi, A. M. Swied

**Affiliations:** ^1^Gastroenterology Division, Department of Medicine 1443, King Abdulaziz Medical City, P.O. Box 22490, Riyadh 11426, Saudi Arabia; ^2^Department of Medicine 1443, King Abdulaziz Medical City, P.O. Box 22490, Riyadh 11426, Saudi Arabia; ^3^Gastroenterology Division, Department of Medicine, King Fahad Medical City, P.O. Box 59046, Riyadh 11525, Saudi Arabia

## Abstract

Tuberculosis as a cause of obstructive jaundice is a rare entity with only a few cases reported in the literature. Patients with this condition usually present with a protracted illness, jaundice, and weight loss, which may be confused with malignancies. We are reporting unusual case of isolated enlarged tuberculous lymph node compressing the common bile duct in the retropancreatic region and causing obstructive jaundice in an immunocompetent patient which to the best of our knowledge is the first case of isolated retropancreatic tuberculous lymphadenitis in Saudi Arabia.

## 1. Introduction

The extrapulmonary tuberculosis (EPTB) represents around 10–25% of all TB cases and this proportion varies between countries. EPTB as cause of obstructive jaundice is a rare entity with only a few cases reported in the literature. Patients with this condition usually present with a protracted illness, jaundice, and weight loss, which may be confused with malignancies.

## 2. Case Report

Our patient is 20-year-old male who is healthy before complaining of a gradual onset right upper quadrant abdominal pain for one month. It was associated with nausea, vomiting, and decreased appetite, yellowish discoloration of the sclera, itching, pale stool, and dark urine. There was no fever, weight loss, or change in bowel habits. There was no history of gallstones or similar previous episodes. He gave a history of contact with a pulmonary TB patient (his aunt) 4 years ago without precaution. His past medical and surgical histories were unremarkable. There was no family history of liver, pancreatic disease, or malignancies. He denied any history of smoking, alcohol consumption, drug abuse, or high-risk sexual exposure.

The patient initially was investigated in another hospital, where he was labeled as a case of a peripancreatic mass with common bile duct compression, and endoscopic retrograde cholangiopancreatography (ERCP) with sphincterotomy and insertion of a plastic biliary stent were done three weeks before seeking our opinion.

In our hospital, he was well, alert, and oriented, not jaundiced; his body mass index was 29.7. Abdominal examination showed soft abdomen and no palpable lymph node or masses. Chest examination was unremarkable.

His complete blood count was normal. Biochemical investigation revealed raised total bilirubin (20.8 mmol/L) with conjugated hyperbilirubinemia (13.6 mmol/L) and raised hepatic enzymes, including AST (57 IU/L), ALT (131 IU/L), and alkaline phosphatase (103 IU/L). All the viral markers and tumor markers (CA 19.9, AFP, and CEA) were negative. ESR was 28 mm/hr and tuberculosis quantiferon was positive.

**Figure 1:**
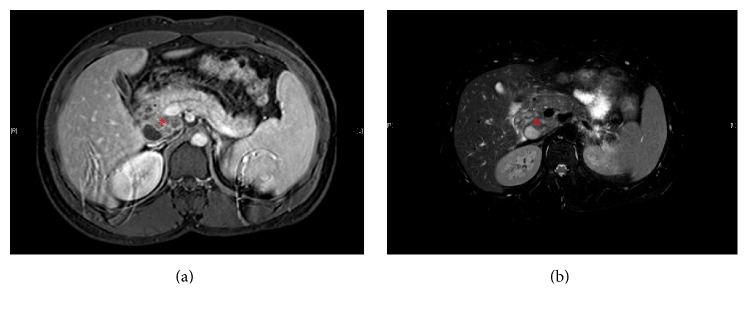
Axial abdominal MRI. (a) T1 MRI with contrast in arterial phase and (b) T2 MRI. Demonstrating a multiloculated complex cystic mass at the posterior aspect of the pancreatic head with heterogeneous content and enhancing septations (asterisk). It measures 4.7 × 4 cm with mild mass effect on the portal vein and no definite communication with common bile duct or pancreatic duct.

Abdomen ultrasound showed hypoechoic peripancreatic soft tissue mass with minimal vascularity measuring 4.8 × 3.2 cm, no pancreatic mass, and common bile duct stent seen likely relieving obstruction caused by the mass; there is no intrahepatic duct dilatation. Abdomen MRI showed complex cystic mass at the posterior aspect of the pancreatic head with no intra-abdominal lymphadenopathy (Figure 1). Chest X-ray was normal.

**Figure 2:**
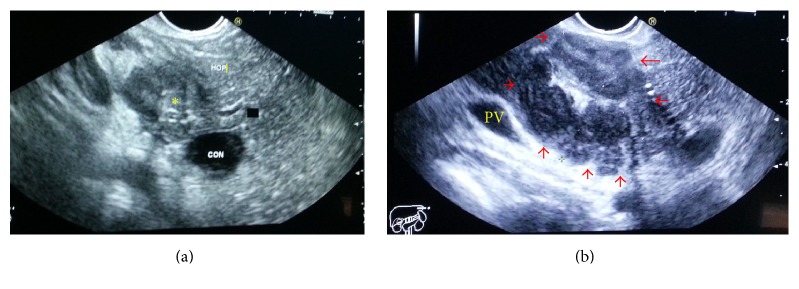
EUS showed a large hypoechoic mass mixed with hyperechoic spots and septate, measuring 4 × 4.7 cm, sets in the retropancreatic area with no fluid content noted with significant mass effect on the common bile duct. Superior mesenteric artery visualized and portal vain patent.

The patient subsequently underwent endoscopic ultrasound (EUS) with fine needle aspiration which showed large hypoechoic mass mixed with hyperechoic spots and septations, measuring 4 × 4.7 cm, sets in the retropancreatic area (Figure 2).

**Figure 3:**
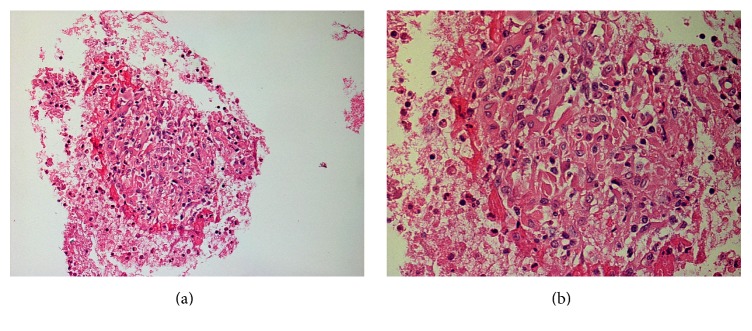
The histological finding of hematoxylin and eosin staining. (a) Low power sections (100x) and (b) high power section (400x) revealed mixed population of lymphoid cells. In addition, there are aggregates of epithelioid cells, forming granulomas; some showed central necrosis occasional multinucleated giant cells are also seen. On immunohistochemistry, the lymphoid cells are a mixture of both B and T lymphocytes forming germinal centers. No Reed-Sternberg cells are seen.

Cytopathological examination showed necrotizing granulomatous lymphadenitis. No fungi or acid-fast bacilli on special staining were seen. TB PCR for* M. tuberculosis* complex was positive (Figure 3).

Antituberculous therapy with isoniazid, rifampicin, pyrazinamide, and ethambutol regimen was initiated. In follow-up, he has gained 4 kg of his weight and normalized his liver enzymes after one month of treatment. Patient completed 9 months of anti-TB therapy without complication, stent then was removed, and his bilirubin level stayed within the normal range in subsequent follow-up.

## 3. Discussion

Tuberculosis (TB) is a major global health problem, causing morbidity and mortality. Worldwide, it is the second leading cause of death from an infectious disease. The extrapulmonary TB represents around 10–25% of all TB cases and this proportion varies between countries [1].

Abdominal tuberculosis is a common extrapulmonary TB site and known to be a diagnostic challenge. It can involve the gastrointestinal tract, peritoneum, lymph nodes, or any intraabdominal organ [1]. The most frequent form of abdominal TB is tuberculous lymphadenopathy, which is related to the disease nature of spreading through lymphatic system [2].

The mesenteric, periportal region and peripancreatic lymph node are commonly involved [2]. TB rarely involves the pancreas [3]. Pancreatic and peripancreatic tuberculosis are difficult to diagnose and often misdiagnosed as pancreatic carcinoma.

The initial symptoms of abdominal TB are variable and depend on the area involved. Commonly patients presented with abdominal pain followed by fever and weight loss. Jaundice was reported in less than thirty percent of the cases [4] and usually it is secondary to involvement of the head of the pancreas, compression of the bile duct by enlarged lymph nodes in the porta hepatis, tubercular stricture of the biliary tree, or a tubercular mass of the retroperitoneum [5].

The definitive diagnosis of abdominal tuberculous lymphadenopathy is based on histopathological and microbiological evidence of tuberculosis. It is usually seen by abdominal CT as hypodense mass or masses with peripheral enhancement, with irregular borders and a multilocular appearance [6]. This is highly suggestive of TB but is not pathognomonic feature.

Recently, endoscopic ultrasound (EUS) has emerged as a safe and accurate tool for imaging and sampling of intra-abdominal pathology. It can differentiate pancreatic and peripancreatic masses as well as identifying abdominal lymphadenopathy that may have been missed on cross-sectional imaging. In addition, EUS guided fine needle aspiration and biopsy can also be performed [7].

In our case, EUS has a major role in the diagnosis. It allows us to assess and visualize the mass adequately and distinguish it from the surrounding structures. In addition to that, we are able to obtain a tissue biopsy in the same setting and avoid unnecessarily invasive surgical procedure.

The diagnostic accuracy of EUS-FNA of pancreatic/peripancreatic TB has been determined in several studies. In a series of 21 consecutive patients with pancreatic/peripancreatic TB EUS-FNA showed 13 (61.9%) had granulomatous inflammation on histopathological examination, 66.7% were positive on a TB PCR assay, Ziehl-Neelsen staining was positive in 26.7%, and 37.5% had positive cultures for* Mycobacterium tuberculosis* [8].

The classical cytological patterns of TB lymphadenitis are Langerhans giant cells, caseating necrosis, granulomatous inflammation, and calcification [9].

Multiple studies have shown that the cytological patterns influence the positivity of AFB stain. In a series of three hundred and eighteen lymph nodes aspirate of clinically suspected tuberculous patients showed the highest AFB positivity was seen in smears revealing necrosis without epithelioid granulomas (85.5%) while the lowest was seen in smears showing epithelioid granulomas without necrosis (3.2%) [9]. Similar result is shown by Bezabih et al. [10].

Drug treatment is the most important modality and follows standard regimens and principles. Six to nine months of therapy is probably adequate for most sites. Most of the pancreatic and peripancreatic tuberculosis cases respond well to antituberculous treatment [4]. The most common regimen was isoniazid, rifampicin, pyrazinamide, and ethambutol [4].

## 4. Conclusion

Isolated peripancreatic tuberculous lymphadenitis is extremely rare. It is a diagnostic challenge and for that a high index of suspicion is needed and should be considered in the context of the peripancreatic or pancreas head mass in the endemic area especially in a young adult.
